# Wilms Tumor With Raised Serum Alpha-Fetoprotein: Highlighting the Need for Novel Circulating Biomarkers

**DOI:** 10.1177/10935266231213467

**Published:** 2023-12-14

**Authors:** Rebecca Green, Adeeb Ahmed, Ben Fleming, Anna-May Long, Sam Behjati, Jamie Trotman, Patrick Tarpey, James C. Nicholson, Nicholas Coleman, C. Elizabeth Hook, Matthew J. Murray

**Affiliations:** 1 Department of Paediatric Haematology and Oncology, Cambridge University Hospitals NHS Foundation Trust, Cambridge, UK; 2 Department of Paediatrics, Norfolk and Norwich University Hospitals NHS Foundation Trust, Norwich, Norfolk, UK; 3 Department of Radiology, Cambridge University Hospitals NHS Foundation Trust, Cambridge, UK; 4 Department of Paediatric Surgery, Cambridge University Hospitals NHS Foundation Trust, Cambridge, UK; 5 Wellcome Trust Sanger Institute, Hinxton, Cambridge, UK; 6 East Genomics Laboratory Hub (GLH) Genetics Laboratory, Cambridge University Hospitals NHS Foundation Trust, Cambridge, UK; 7 Department of Paediatrics, Level 8, Cambridge University Hospitals NHS Foundation Trust, Cambridge, UK; 8 Department of Paediatric Histopathology, Cambridge University Hospitals NHS Foundation Trust, Cambridge, UK; 9 Department of Pathology, University of Cambridge, Cambridge, UK

**Keywords:** alpha-fetoprotein, biomarker, microRNA, Wilms tumor, WT

## Abstract

Wilms tumor (WT) is the commonest cause of renal cancer in children. In Europe, a diagnosis is made for most cases on typical clinical and radiological findings, prior to pre-operative chemotherapy. Here, we describe a case of a young boy presenting with a large abdominal tumor, associated with raised serum alpha-fetoprotein (AFP) levels at diagnosis. Given the atypical features present, a biopsy was taken, and histology was consistent with WT, showing triphasic WT, with epithelial, stromal, and blastemal elements present, and positive WT1 and CD56 immunohistochemical staining. During pre-operative chemotherapy, serial serum AFP measurements showed further increases, despite a radiological response, before a subsequent fall to normal following nephrectomy. The resection specimen was comprised of ~55% and ~45% stromal and epithelial elements, respectively, with no anaplasia, but immunohistochemistry using AFP staining revealed positive mucinous intestinal epithelium, consistent with the serum AFP observations. The lack of correlation between tumor response and serum AFP levels in this case highlights a more general clinical unmet need to identify WT-specific circulating tumor markers.

## Introduction

Wilms tumor (WT), also known as nephroblastoma, is the most common renal tumor in childhood; 95% of cases are diagnosed under 10 years of age, with a median age of three years.^1^ Histologically, WT is classically characterized by a triphasic appearance with three cell types typically seen; namely blastemal, stromal, and epithelial cells, however, monophasic and biphasic WT are also commonly seen.^2^ WT can also have areas of diffuse anaplasia,^3^ an unfavorable histological feature. Rarely, ‘teratoid’ WT may be described,^4^ referring to WT containing teratoid features with marked heterologous differentiation. In North America, the Renal Tumor Committee of the Children’s Oncology Group (COG-RTC) recommends primary nephrectomy and subsequent treatment based on histology, stage, and biology. In contrast, standard practice in Europe from the International Society of Paediatric Oncology (SIOP) is to diagnose WT based on clinical and radiological findings, followed by neo-adjuvant chemotherapy, prior to nephrectomy.^5-7^ Unfortunately, there are no current circulating biomarkers to assist diagnosis and disease-monitoring for WT in routine clinical practice. Of note, WT is very rarely associated with raised serum alpha-fetoprotein (AFP),^8^ a tumor marker typically raised in primary liver tumors such as hepatoblastoma and germ cell tumors (GCT).^9^ Here, we describe a case of a young child presenting with a large WT associated with raised serum AFP levels. This case demonstrates some of the clinical diagnostic challenges with pediatric abdominal tumors and the need for more specific circulating biomarkers for WT.

## Case Report

**Figure 1.**
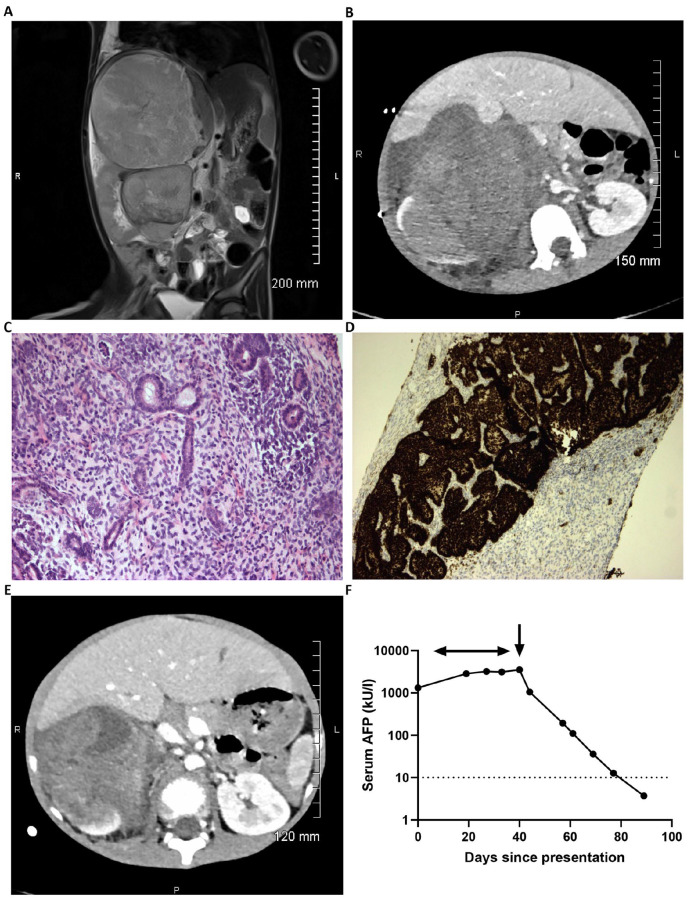
Initial radiological, pathological, and biochemical findings in the Wilms tumor case. (A) Representative T2 coronal slice of the diagnostic MRI scan demonstrating the bilobed tumor with larger superior component and apparent claw sign with the right lobe of the liver and a smaller inferior component and apparent renal claw sign. (B) Representative axial image from the subsequent diagnostic CT scan demonstrating the large intra-abdominal tumor and presence of a renal claw sign. (C) and (D) Representative pathology of the initial biopsy specimen. (C) Histological image showing triphasic WT with blastemal, stromal, and epithelial elements (×200). (D) WT1 immunohistochemical staining showing positive expression by blastemal and epithelial elements with negative staining of stromal elements (×100). (E) Repeat CT scan following four weeks of neoadjuvant ‘VA’ chemotherapy showing reduction in tumor size. (F) Graph demonstrating log_10_ serum AFP values from clinical presentation (day 0) until normalization at day 89. The horizontal arrow represents timing of delivery of pre-operative chemotherapy; vertical arrow represents timing of surgical resection of the WT on day 40.

A 5-year-old boy presented with a short history of fever, vomiting, abdominal pain, and distension. On clinical examination, he was hypertensive with a mass palpable in the right upper quadrant. There were no clinical features to suggest an associated syndrome. Initial movement degraded MRI confirmed a large heterogeneous bilobed tumor with a larger superior component centered on the right lobe of the liver with an apparent claw sign and a smaller inferior component centered at the level of the right renal hilum with an apparent renal claw sign. Subsequent CT showed a more convincing renal claw sign, while the apparent claw of liver tissue seen around the lesion on MRI appeared to be tumoral (Figure 1(A) and (B)). Staging CT chest showed no metastatic lung disease. Blood tests were unremarkable, save for a raised serum lactate dehydrogenase (LDH) of 929 units/L (upper limit of normal 368 units/L) and raised serum AFP of 1325 kU/L (<10 kU/L); urine catecholamines were normal. The AFP was performed due to the radiological possibility of a primary liver tumor or hepatic GCT.^9^ Given the diagnostic uncertainty, ultrasound-guided percutaneous biopsy of the mass was performed, as recommended.^5^ Histology from the biopsy showed a triphasic WT (Figure 1(C)), with no evidence of anaplasia. Immunohistochemistry showed patchy but definitive staining with WT1 (Figure 1(D)) and CD56; INI-1 expression was retained. He received four weeks of pre-operative ‘VA’ chemotherapy with vincristine and actinomycin D as per the current UK UMBRELLA protocol.^7^

Reassessment CT scan four weeks into pre-operative chemotherapy showed a substantial reduction in tumor size from 15.7 × 14.5 × 22.8 cm at diagnosis (tumor volume 2715 cm^3^) to 10.5 × 10.7 × 15.0 cm (881 cm^3^; Figure 1(E)), where the volume was the product of the three tumor diameters × 0.523. Conversely, the serum AFP continued to rise, peaking at 3549 kU/L at this time. Following radical nephrectomy, the AFP level fell to 1051 and 194 kU/L at post-operative days 3 and 17, respectively, normalizing (<10 kU/L) 7 weeks following surgery (Figure 1(F)).

**Figure 2.**
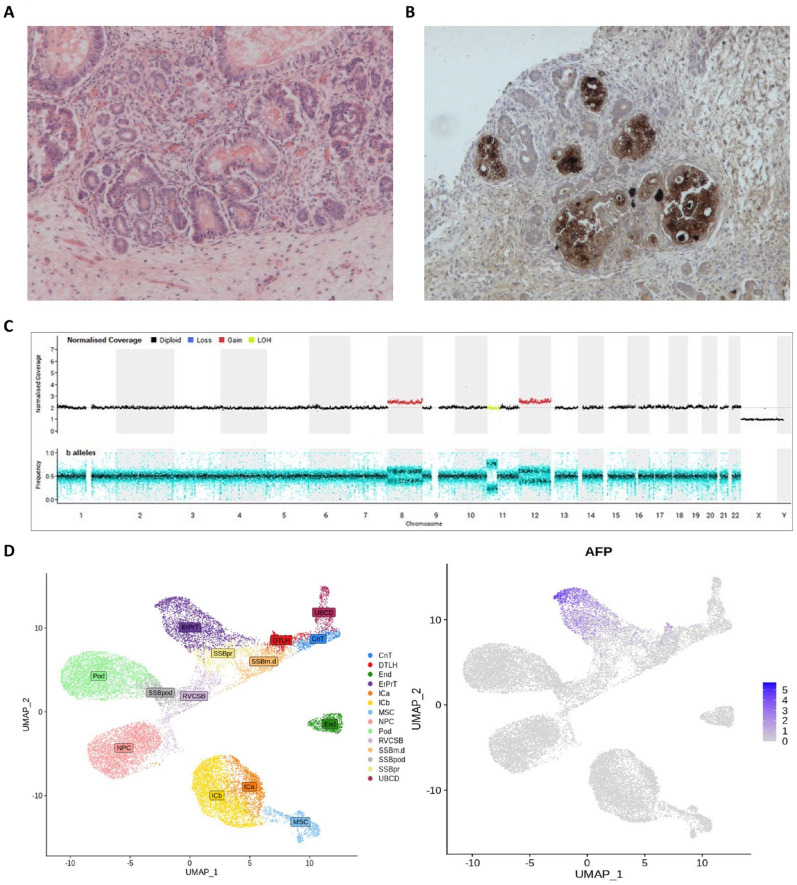
Histopathological features of the resected Wilms tumor. (A) and (B) Representative pathology of the resection specimen after induction chemotherapy. A) Histological image showing prominent residual stromal and epithelial elements (×200). (B) AFP immunohistochemical staining showing foci of positive expression by heterologous mucinous intestinal epithelial elements (×200). (C) Whole genome sequencing copy number profile (top-panel) and b-allele ratio (bottom-panel) illustrating trisomy of chromosomes 8 and 12 (red normalized coverage) and copy number neutral loss of heterozygozity (LOH) on chromosome 11p (yellow normalized coverage). (D) Uniform Manifold Approximation and Projection for Dimension Reduction (UMAP) images showing normal fetal kidney cell type annotation (left) and expression of AFP in the early proximal convoluted tubule (ErPrT) during renal development (right). Abbreviations: Annotation Key: CnT, connecting tubules; DTLH, distal tubule and loop of Henle; End, endothelium; ErPrT, early proximal tubules; Ica, interstitial cells a (smooth muscle); ICb, interstitial cells b (stromal); MSC, mesenchymal stem cells; NPC, nephron progenitor cell (cap mesenchyme); Pod, podocytes; RVCSB, renal vesicle and comma-shaped body; SSBm.d, S-shaped body medial and distal; SSBpod, S-shaped body podocytes; SSBpr, S-shaped body proximal tubules; UBCD, ureteric bud and collecting duct.

Histology of the resection specimen revealed substantial areas of necrosis affecting approximately one third of the tumor volume. The viable tissues showed a mixed composition. Approximately 55% was comprised of stromal elements, with some foci of heterologous cartilage and skeletal muscle present, and ~45% epithelial elements, with a variety of mucinous glandular arrangements (Figure 2(A)). There were also small regions of blastema, representing 1% of the viable tumor. There was no evidence of anaplasia within the resected specimen, but immunohistochemistry using AFP staining revealed positivity in ~10% of the mucinous intestinal epithelium (Figure 2(B)). There was viable tumor extending to the resection margins and attenuation of the renal capsule peripherally. Resected lymph nodes showed reactive changes only. Whole genome sequencing (WGS) was also offered and undertaken as standard-of-care testing on the original pre-chemotherapy biopsy specimen. This revealed a quiescent genome with a low tumor mutation burden (approximately 1 non-synonymous mutation/Mb coding sequence). A missense variant in MAPK1 Asp321Ala, recurrent in diverse cancer types, was the only likely driver. Mutational signatures were predominantly age-related. Copy number variation included copy-neutral loss of heterozygosity (LOH) on chromosome 11p consistent with Wilms tumor, and trisomy of chromosomes 8 and 12 (Figure 2(C)). No structural variants were identified. In particular, there were no changes on chromosome 4 where the AFP gene resides. No other molecular biomarker studies were performed in this single case, such as microRNA testing or circulating tumor DNA (ctDNA) assessment (see Discussion section).

Overall, the conclusion was an abdominal stage III WT, intermediate risk with mixed type histology, according to the current UK UMBRELLA pathology guidelines.^6^ Accordingly, the patient received six months of post-operative VA chemotherapy and abdominal radiotherapy^7^ and remains well 21 months following presentation, and 14 months following end of treatment.

## Discussion

We present a 5-year-old boy with an abdominal mass in whom the initial radiological appearances were diagnostically challenging. In clinically and radiologically typical cases, where the mass is clearly of renal origin, neither SIOP nor the COG-RTC recommend biopsy due to the very high prevalence of such tumors representing WT.^5^ However, if there are features that are atypical for WT, then a diagnostic biopsy is recommended. In this case, there was doubt about the organ of origin of the tumor based on the initial MRI, and also atypical biochemical features, namely high serum LDH and AFP, further contributing to the diagnostic uncertainty. It was therefore prudent to obtain a diagnostic biopsy prior to commencing definitive treatment.

The case described here highlights an unusual association of WT with raised serum AFP. AFP is a serum protein that is produced by the fetal liver and the yolk sac. It is therefore typically elevated in primary hepatic tumors such as hepatoblastoma, or tumors with yolk sac elements, such as malignant GCTs.^9^ There are a small number of case reports of WT associated with raised serum AFP,^10^ including those with (marked) heterologous differentiation,^4^ previously termed ‘teratoid’ variants. Two single case studies report a reduction of serum AFP levels with pre-operative chemotherapy.^10,11^ A series of three cases demonstrated a reduction of serum AFP in response to treatment with chemotherapy and surgery with a subsequent rise in AFP related to disease progression in one of the patient.^8^ In another study, a further three WT cases were described associated with high serum AFP at diagnosis, which remained high throughout pre-operative chemotherapy. However, this was associated with lack of tumor regression.^12^ Amongst these reported cases, serum AFP levels fell following surgery, as observed here. The novel feature in our case was the marked rise in serum AFP during pre-operative chemotherapy, despite a substantial reduction in tumor size, both clinically and radiologically. Serum AFP levels were therefore not useful for disease-monitoring purposes here.

The prolonged elevated serum AFP level observed here across the 4-week period prior to surgery has a number of potential explanations, which are not necessarily mutually exclusive. In terms of AFP origin, the histological features seen in the tumor resection specimen, with AFP-positive mucinous intestinal epithelium, may well explain the high serum AFP observed here. Further interrogation of previously published data revealed additional novel insights, namely that these observations are also consistent with the presence of AFP positive early proximal convoluted tubules (ErPrT) of the human fetal kidney, which WT is thought to recapitulate^13^ (Figure 2(D)). Regarding persistence, the continued AFP rise seen pre-operatively may have been due to a ‘flare.’^14^ Such a flare is typically a short-lived (1-2 week) AFP rise following initiation of treatment, and may be seen in patients with malignant GCTs, followed by the expected decline. The phenomenon is believed to derive from acute AFP released from tumor cells killed by chemotherapy, rather than representing a lack of disease response. Here, the AFP rise may have represented more sustained ongoing tumor kill from weekly chemotherapy, in a tumor type which is generally less exquisitely chemosensitive compared with GCTs, and which therefore typically requires longer treatment courses than those used for cure for patients with GCTs. As expected, following tumor resection, the AFP level fell rapidly to normal once the source of production had been removed.

These observations highlight that identification of specific circulating biomarkers for patients with WT remains an urgent unmet clinical need. Such identification would facilitate diagnosis and disease-monitoring, and may reduce the need for upfront biopsy and/or serial imaging. MicroRNAs (miRNAs) are short, non-coding RNA molecules that have shown promise as circulating cancer biomarkers in tumors which affect children, such as neuroblastoma.^15,16^ Studies investigating circulating miRNAs in patients with WT have identified candidate biomarker lists for further interrogation,^15,17^ recently reviewed elsewhere.^18^ Furthermore, circulating miRNA signatures appears independent of pre-operative chemotherapy treatment.^19^ Given the heterogeneity of WT, a formal robust consensus diagnostic panel of miRNAs for WT has not yet been established, and therefore we did not pursue circulating miRNA testing in our single patient here. However, large prospective studies, such as under the UMBRELLA study, will aim to establish such a signature. Of note, mutations in the tumor suppressor gene *TP53* have also been identified in ctDNA in the bloodstream of patients with diffuse anaplastic WT.^20^ It will be interesting to discover whether any such miRNAs or ctDNA detectable/elevated in the bloodstream at diagnosis are also secreted and detectable at elevated levels in the urine, making any putative assay even more non-invasive. Further studies will be required to extend and implement these initial biomarker discoveries, which have important potential clinical benefits.

In summary, we describe an unusual case of WT, associated with substantially raised serum AFP at diagnosis which rose further during pre-operative chemotherapy, and fell rapidly upon tumor resection. The case highlights some of the challenges in diagnosing pediatric intrabdominal tumors, especially when they present atypically, and also demonstrates the lack of correlation between AFP and response to chemotherapy in WT. This report also highlights that specific WT markers are urgently required to facilitate more accurate and timely diagnosis, disease-monitoring, and follow-up for such patients, with the ultimate aim of improving clinical outcomes.
